# Transepithelial Photorefractive Keratektomy after a Clear Lens Exchange

**DOI:** 10.3390/vision5010008

**Published:** 2021-02-03

**Authors:** Diego de Ortueta

**Affiliations:** AURELIOS, Augenlaserzentrum Recklinghausen, Erlbruch 34-36, 45657 Recklinghausen, Germany; Diego.de.Ortueta@augenzentrum.org; Tel.: +49-2361-3069770; Fax: +49-2361-3069799

**Keywords:** TransPRK, smart pulse, Smartsurface, PRK, IOL, touch up, EDOF, multifocal, AMARIS, ametropia, astigmatism, hyperopia, myopia

## Abstract

Purpose: We evaluated the refractive visual outcomes and efficacy of Transepithelial Photorefractive Keratectomy (TransPRK) using Smart Pulse Technology with static and dynamic cyclotorsion and the AMARIS 1050 Hz RS laser platform from Schwind in the eyes after a refractive lens exchange. Setting/Venue: Aurelios Augenlaserzentrum, Recklinghausen. Methods: We retrospectively evaluated the data of 552 consecutive eyes treated with refractive lens exchange between 2016 and 2019. A total of 47 eyes (8.5%) required a touch up after the clear lens exchange. From 43 eyes of 43 patients, we obtained a minimum follow up of 3 months. In all cases, we performed a TransPRK with a minimum optical zone of 7.2 mm, centering the ablation on the vertex of the cornea. Results: The average age of the treated eyes was 57 years old, with a range between 48 and 68 years. The mean treated sphere was 0.42 diopters (D), with a range between −1.0 and +1.75 D. The mean astigmatism was 1.06 D. Postoperatively, after laser vision correction, we reduced the sphere to a mean of 0.11 D (range −0.5 to +0.75 D), and, postoperatively, the mean astigmatism was 0.25 D (range −0.75 to 0 D). The predictability for a spheric equivalent (SEQ) of 0.5 D was 91%, and for 1 D it was 100% of the cases. No eye lost more than one Snellen line. Conclusions: TransPRK with smart pulse was predictable for correcting ametropia after Clear Lens Surgery.

## 1. Introduction

Residual refractive errors are not tolerated in patients treated with refractive lens exchange and premium lenses; to maximize patient satisfaction and obtain a good postoperative uncorrected visual acuity (UCVA), we need to perform, in some cases, a second surgery. We can change the implanted intraocular lens or add a second intraocular lens (piggyback method) [1,2]; these are options if the ametropia is very large; a third alternative is to use refractive corneal surgery. Laser vision correction (LVC) has been shown to be predictable and effective for correcting small amounts of spherical and cylindrical errors after intraocular lens surgery [3,4,5,6].

The principal cause of patient dissatisfaction after premium lens implantation is a bad uncorrected visual acuity. Despite a new generation of intraocular lens power calculation formulas [7], small amounts of astigmatism (>0.5 diopters (D)), hyperopia (>0.25 D) or myopia (>0.5 D) can occur and cause a bad UCVA.

The aim of this study was to evaluate the efficacy of correcting the residual refractive error using transepithelial photorefractive keratectomy (TransPRK) after clear lens exchange (CLE). There are similar studies analyzing the results of photorefractive keratectomy (PRK) [3,4,5,6] or laser in situ keratomileiusis (LASIK) [8] after cataract surgery. In our opinion, this is the first study analyzing eyes treated with clear lens exchange followed by the TransPRK method.

TransPRK techniques are an advancement over conventional laser-assisted subepithelial keratectomy (LASEK) and PRK, delivering a more sophisticated no-touch all-laser single-step refractive procedure [9,10,11]. In transepithelial PRK, refractive errors are treated by superimposing a defined epithelial thickness profile (approximately 55 µm at the center and 65 µm at the periphery, 4 mm radially from the center in the Schwind AMARIS platform lasers, Kleinostheim, Germany) with a corneal aspheric ablation profile. The terminology ‘‘reversed single-step’’ outlines the pseudo-sequentialization of the corneal aspheric profile, and the epithelial thickness profile components are realized in a single step without breaks. This means that, in a counterintuitive way, the refractive correction is applied first and the epithelial profile at the end [9]. Many groups have reported the efficacy and safety of TransPRK procedures [10,11,12,13,14].

## 2. Methods

### 2.1. Patients

This retrospective cohort study was based on a retrospective consecutive case series of patients treated by a single surgeon (DdO) with TransPRK to correct ametropia after refractive lens exchange, at Aurelios Augenlaserzentrum, Recklinghausen, Germany. The study followed the tenets of the Declaration of Helsinki. The institutional review board approved this retrospective evaluation. Proper informed consent was obtained from each patient, for both the treatment and use of their de-identified clinical data for publication.

The inclusion criteria for the study were: patients older than 40 years, treated with CLE, who had a refractive error without systemic disease with ocular involvement. Those with concurrent ocular disease, severe dry eye, previous corneal or ocular surgery other than the lens surgery, keratoconus suspects or subclinical or clinical keratoconus were excluded from the study.

The retrospective study analyzed eyes treated from January 2016 till December 2019. We performed, in this time, 552 clear lens exchange surgeries. From this group, 47 eyes (8.5%) required a correction of the refractive residual error. We had a minimum follow up of 3 months in 43 eyes of 34 patients.

The average treated age was 57 years old with a range between 48 and 65 years and a standard deviation (SD) of 7 years. The mean preoperative sphere was 0.42 diopters (D) (a range between −1 D and +1.75 D), and the mean cylinder was −1.06 D with a range to −3.25 D. In two eyes, we preoperatively planned a LVC after the clear lens exchange because the cylinder was preoperatively higher than 2 diopters, and we decided to implant a diffractive multifocal lens without correcting the astigmatism intraoperatively. In all other cases, the LVC was not planned. The causes to perform LVC after CLE were a manifested astigmatism of >0.5 D in 25 of the eyes (58%), a hyperopia of >0.25 D with <0.75 D astigmatism in 8 cases (19%), and a myopia >0.5 D with <0.75 D astigmatism in 10 cases (23%).

We performed CLE with a clear cornea incision and phacoemulsification with the Alcon Centurion machine (Fort Worth, TX, USA) in all the cases without complications.

The manually performed corneal incision was in the steepest meridian. Two fellow eyes were operated on at least 1 week apart. For the intraocular lens (IOL) power calculation, the Haigis formula was used according to the measurements of axial length, corneal power, and anterior chamber depth measured by the IOLMaster 500 (Carl Zeiss Meditec, Jena, Germany) in all cases. The type of lenses that required a laser LVC were, in 19 cases, the ZLB00 (Johnson & Johnson, New Brunswick, NJ, USA), a diffractive bifocal lens with an addition of +3.25 D; three eyes had a ZMB00 (Johnson & Johnson, New Brunswick, NJ, USA) implanted, a diffractive bifocal lens with an addition of 4 D; seven eyes had a ZXR00 (Johnson & Johnson,) New Brunswick, NJ, USA with an extended depth of focus; seven eyes had a ZXT00 (Johnson and Johnson, New Brunswick, NJ, USA) toric lens with an extended depth of focus; three eyes had a ZCT (Johnson & Johnson, New Brunswick, NJ, USA), and four eyes had the POD L GF (Physiol, Liege, Belgium), a trifocal lens with additions of 1.75 and 3.5 D.

### 2.2. Preoperative Assessment

A full ophthalmologic examination was performed on all the patients prior to surgery, including manifest refractions, slit-lamp microscopy of the anterior segment, corneal-wavefront analyses (Sirius, CSO, Florence, Italy), and ocular wavefronts measured using a commercial pyramid wavefront sensor (Peramis; SCHWIND eye-tech-solutions, Kleinostheim, Germany). To plan the refractive correction, we used the refractive data of the ocular wavefront at 4 mm. Starting with this refraction, we performed a monocular subjective refraction. For the astigmatism, we also used the data of the simulated keratometry of the topography and the ocular wavefront at 4 mm, which is the most widely accepted astigmatism analysis for far and near vision.

The corrected distance visual acuity (CDVA) and uncorrected distance visual acuity (UDVA) were assessed with EDTRS charts. The corrected visual acuity was always assessed with trial frames. As we decided only to treat with LVC after CLE first, only one eye presented the correction at the right or left eye without correcting the fellow eye and showing the advantage and disadvantages of the correction for near and far vision. Only eyes showing better vison with correction were then treated. Eyes with extended depth of focus intraocular lenses with a myopic residual error may have worse visual distance acuity but better near visual acuity, and it may be that the best visual satisfaction result is achieved with one eye near emmetropia and the other one with a residual myopia.

### 2.3. Surgical Procedure

The TransPRK is a one-step procedure with a no-touch technique, superimposing an aspheric PRK profile with an aspheric Photorefraktive Keratektomy (PTK) for the epithelium. We used TransPRK with smart pulse technology (SPT, Schwind eye-tech. solutions, Kleinostheim, Germany)) [13,14]. The normal ablation profile describes a 3-dimensional volume based on a flat corneal surface, whereas with the SPT profile, the volume is based on a curved corneal surface using a fullerene structure, which means that each ablation point is equidistant.

Aspheric non-wavefront-guided treatments were performed in all cases. For each treatment, the Schwind CAM (Schwind eye-tech.solutions, Kleinostheim, Germany) planning software calculated the size of the optimal transition zone, depending on the preoperative refraction and optical treatment zone (OZ). We used a large OZ of 7.2–7.5 mm, as the amount of correction was very small.

Patients received one drop of Diclofenac (0.93 mg pro mL) and took 600 mg Ibuprofen 20 min before surgery. One drop of topical anesthetic Oxybuprocain (1.79 mg pro 0.5 mL) was instilled in the upper and lower fornices just before entering the surgical room. A second drop of topical anesthetic was instilled after aligning the patient onto the patient table. A lid speculum was inserted to allow maximum exposure of the globe; a third drop of topical anesthetic was instilled, and 1 mL cold (10 °C) balanced salt solution was applied before surgery.

All surgeries were performed with the Amaris excimer laser, 1050RS (SCHWIND eye-tech-solutions, Kleinostheim, Germany). Proper alignment of the eye under the laser was achieved with a 1050 Hz infrared eye tracker with simultaneous limbus, pupil, and torsion tracking integrated into the laser system and centered on the corneal vertex using the pupillary offset [15] determined by the topographer, which closely approximates the visual axis [16]. The eye tracker had a typical response time of 1.7 milliseconds with a system total latency time of 2.9 milliseconds. Patients were requested to look at a pulsing green fixation light throughout the ablation.

After the eyes were treated, the interface was irrigated with a balanced salt solution, to remove any debris. At the end of the procedure, one Ofloxacin drop (3 mg pro mL), one Dexamethason drop (0.5 mg pro 0.5 mL), and one Phenylephrin drop (82.1 mg pro mL) were applied, and bandage contact lenses were placed before discharging the patient. The patients took IsoptomMax (Novartis Pharma Gmbh, Basel, Switzerland) eye drops four times a day for 2 weeks, Fluormetholon (Fluoropos Ursapharm GmbH, Saarbrücken, Germany) three times per day for another 6 weeks, and preservative free lubricants for 2 months as needed and beyond as per the need.

We did not use Mitomycin C for these small corrections.

### 2.4. Postoperative Evaluation

The patients were reviewed at 1 day, 4 days, 1 month, and 3 months post operatively. The postoperative evaluation included the measurement of the monocular UDVA, manifest refraction, monocular CDVA, and topography (Sirius, CSO, Italy). The ocular wavefronts were measured using a commercial pyramid wavefront sensor (Peramis; SCHWIND eye-tech-solutions, Kleinostheim, Germany).

### 2.5. Statistical Analysis

The visual acuity was measured in a decimal scale converted to logMAR and presented in Snellen Equivalents for reporting comparability. The uncorrected and corrected visual acuity, spherical equivalent refraction, and refractive astigmatism were evaluated.

## 3. Results

Postoperatively, after 3 months, we reduced the mean sphere to nearly 0 and the astigmatism to a mean of 0.22 D. Further details are shown in Table 1.

**Figure 1:**
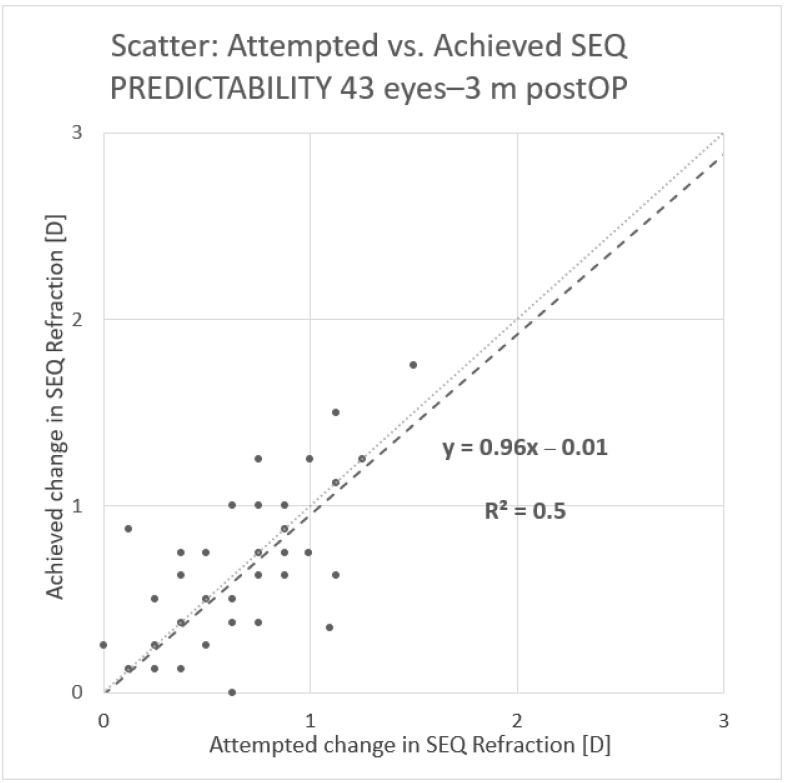
Histogram of predictability. Scatter of the attempted versus achieved spherical equivalents 3 months after the Transepithelial Photorefractive Keratectomy (TransPRK).

**Figure 2:**
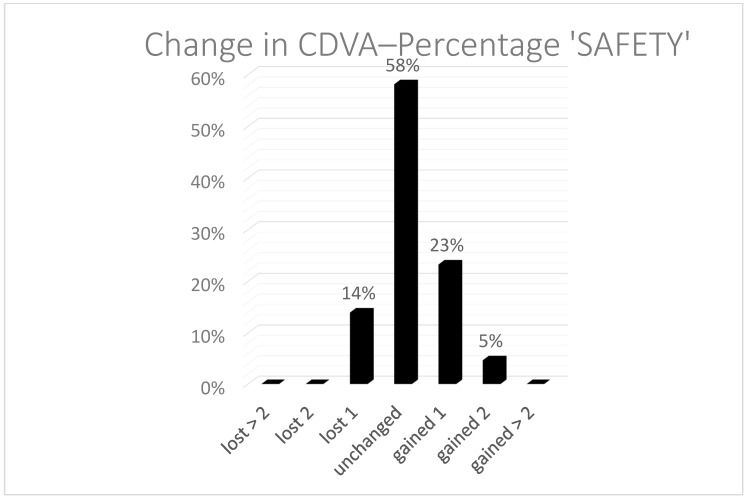
Safety. Comparing lines of vision preoperatively and 3 months postoperatively. No eye lost two or more lines after TransPRK in the eyes treated with clear lens extraction, and 4% of the eyes gained two lines of Snellen acuity.

The predictability is shown the histogram (Figure 1), with a coefficient of regression near to 1. The spherical equivalent refraction (SEQ) was found in 91% of the eyes under a half diopter and 100% under 1 D. In two cases, we performed a TransPRK treatment. One was a hyperopic treatment, and the second was an eye with 0.75 D of astigmatism. After the second TransPRK, the SEQ and astigmatism were under 0.5 D. Safety was also good, with no eyes losing two or more Snellen lines (Figure 2).

**Figure 3:**
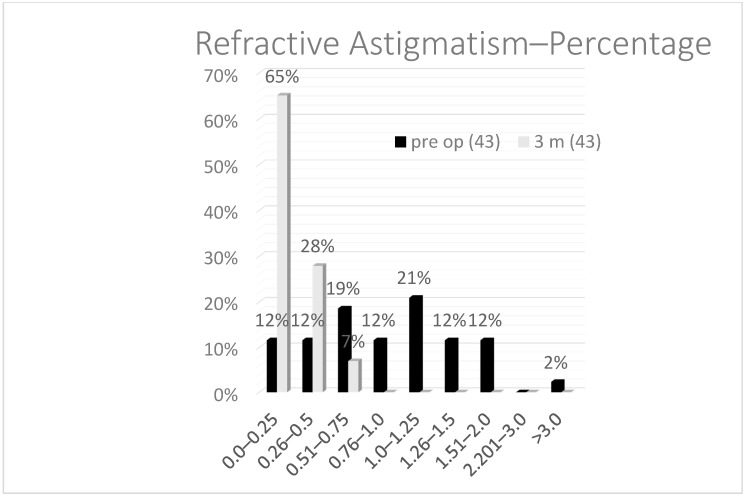
Comparison of the preoperative astigmatism and 3 months after the TransPRK treatment.

As for the astigmatism (Figure 3), which is, in most of the cases, the cause to perform a fine tuning on the cornea after a lens exchange, the mean preoperative astigmatism was 1.2 D and, postoperatively, after 3 months, 0.22 D. Statistically, using the paired Student’s *t*-test, there was a significant reduction after TransPRK of the cylinder and sphere but not the SEQ. This can be explained, as the reason for performing a TransPRK after CLE was astigmatism in most of the cases, and, in these cases, the SEQ was preoperatively near 0.

If we compare the preoperatively best corrected distance visual acuity (BCDVA) with the postoperative uncorrected distance visual acuity (UCDVA), the efficacy was 0.8.

## 4. Discussion

The group of eyes that we analyzed was composed of eyes with preoperatively good visual acuity in which we performed a CLE, and the expectations of this group of patients is high. Residual ametropia greater than 0.50 D was the mean cause for dissatisfaction in these eyes. In most of the cases, residual astigmatism was the cause for performing an LVC. Most of the lenses used were multifocal. In our center, we used lenses with bifocal and trifocal depths of focus, and lenses with extended depth of focus. We observed that the majority of the lenses that needed a LVC were trifocal lenses. Recently, a paper by Seiler et al. [17] showed that trifocal lenses in a population of cataract patients required a laser vision correction in 26% of the cases.

One of the difficulties of using multifocal lenses is to measure the ametropia, and measurements carried out in patients fitted with diffractive intraocular lenses (IOL) may not be reliable. Many aberrometers cannot achieve an accurate measurement through these lenses. The refraction may change depending on the lighting conditions and pupil size in the multifocal IOLs. For spherical subjective refraction, a reference point as the midpoint of the clear vision interval provided by the depth of field of the IOL should be established when refracting patients with multifocal IOLs to avoid postoperative problems of predictability [18]. For the astigmatism, we used the data of the topography and the ocular wavefront and proof, which are the most widely accepted astigmatism measurements for far and near vision.

Photorefractive keratectomy (PRK) is a safe and effective procedure to correct residual refractive errors following cataract surgery with premium IOL [5,19]. We decided to perform a transepithelial approach in our group of eyes. With the TransPRK method, the diameter of epithelial removal was calculated to match the ablation zone, thus decreasing the wound surface and speeding up the healing process in comparison to PRK or LASEK, resulting in a faster visual recovery. A study by Lee et al. [20] reported that the epithelium regrowth was the fastest in the TransPRK-treated group with a mean time of 2.5 days, with 3 days in the PRK group and 3.5 days in the LASEK group.

We also used the smart pulse technology (SPT) software. The SPT software allows for a quicker visual recovery and a smoother surface of the ablated area [21]. A smoother cornea has a potential positive effect on vision, particularly during the first few days after treatment. Vincingerra et al. [22] demonstrated superior results for advanced surface ablation performed with the Amaris 1.050-Hz excimer laser with SPT when compared to the 750-Hz excimer laser, with better uncorrected acuity outcomes and better safety, as well as no eye losing any lines of CDVA at 6 months.

In our 43 cases, the total amount of the corneal higher order aberrations under 6 mm were under 0.3 microns; therefore, we used the aberration free treatment centered on the vertex of the cornea. Aspheric non-wavefront-guided treatments were performed in all cases, i.e., the ablations were optimized to induce no change in the wavefront aberration (within the optical zone, OZ) other than the sphere and cylinder components, leaving all existing high-order aberrations (HOA) unchanged [23]. The induction of new aberrations was minimized [24]. Based on the existing corneal shape and the keratometric values of the cornea, the ideal ablation profile was then calculated, compensating for, among others, the cosine effect [25,26,27].

With the TransPRK, we changed the shape of the epithelium, and, in cases when the epithelium thickness matched with the precalculated thickness of the software, we had a perfect treatment. One of the first papers describing this technique was published by Arba and Awwad [28]. In this theoretical paper, the authors described the possibilities of this technique. TransPRK performed with the AMARIS laser system applies an epithelial thickness profile that resembles a slight hyperopic treatment. Arba and Awwad [28] described very well what happens if the epithelium is thinner or thicker than the values given by the algorithms of the software (55 microns at the center and 65 microns at 4 mm of the periphery).

The achieved optical zone (OZ) is reduced whenever the actual corneal epithelial profile is thicker than the applied epithelial ablation profile. The thicker the corneal epithelial profile, the smaller the achieved OZ. For those cases, the achieved OZ also depends on the amount of refractive correction and the planned OZ. The larger the planned refractive correction, the less the difference between the planned OZ and achieved OZ. Wasted tissue occurs whenever the actual corneal epithelial profile is thinner than the applied epithelial ablation profile. To solve the problem, in treating small amounts of refractive error, which was the case in this group of eyes, we decided to use a larger optical zone of at least 7.2 mm to avoid the problem when the epithelium is thicker than the precalculated value. Since 2020, we now have the ability to introduce the epithelium thickness at the Schwind ORK CAM software (Schwind eye-tech. solutions, Kleinhosteim Germany), and further studies will show if this will produce even better results.

There were three limitations to this work: the retrospective nature of the study imposed a natural limitation on the cohort, the sample size included only 43 eyes, and finally the follow-up was limited to 3 months postoperatively. Despite these limitations, our results show that using TransPRK with Smart Pulse Allocation was suitable for correcting low levels of ametropia after a clear lens exchange.

In conclusion, TransPRK applying smart pulse allocation using a non-wavefront-guided aberration neutral ablation profile yielded excellent visual outcomes for correcting ametropia after refractive lens exchange.

## Figures and Tables

**Table 1:** The preoperative spherical equivalent (SEQ) with standard deviation (SD), sphere (sph), and cylinder (Cyl) compared with the results after 3 months follow-up.

	Preoperative		3 Months Follow-Up	
	Mean ± SD	Range	Mean ± SD	Range
SEQ	−0.11 ± 07.1	−1.13 to +1.5	−0.11± 0.30	−0.75 to +0.38
Sph	+0.42 ± 0.74	−1.0 to +1.75	0.01 ± 0.29	−0.50 to 0.75
Cyl	−1.06 ± 0.62	−3.25 to 0	−0.25 ± 0.25	−0.75 to 0.00

## Data Availability

All data were fully anonymized and are available upon request.
